# Guided growth surgery for angular deformity of the knee: one centres experience

**DOI:** 10.1007/s11845-024-03794-3

**Published:** 2024-08-24

**Authors:** Dave M. Moore, Henry Turner, Oliver Boughton, Maria Noonan, Jacques Noel, Pat Kiely, Pat O’Toole, Paula M. Kelly, David P. Moore

**Affiliations:** Department of Trauma and Orthopaedic Surgery, Children’s Health Ireland, Crumlin, Cooley Rd, Dublin, D12 N512 Ireland

**Keywords:** Angular deformity, Deformity, Genu valgum, Genu varum, Guided growth, Knee

## Abstract

**Background:**

Angular deformity in the lower extremity can result in pain, gait disturbance, cosmetic deformity and joint degeneration. Up until the introduction of guided growth, which has since become the widely accepted treatment for frontal plane angular angular deformity around the knee in skeletally immature patients, treatment consisted of staples, corrective osteotomy or an angular epiphysiodesis. Guided growth modulation uses the tension band principle with the goal of treatment being to normalise the lower limb mechanical axis resulting in lower morbidity than previous treatments. In order to assess the success of this procedure we reviewed our results in an attempt to identify patients who may not benefit from this elegant procedure.

**Methods:**

We performed a retrospective review of prospectively collected surgical records and diagnostic imaging in our paediatric tertiary national referral centre to identify all patients who had guided growth surgery for coronal plane angular deformity of the knee from 2007 to 2023. We noted the patient demographics, diagnosis, peri-operative experience and outcome. All patients were followed until skeletal maturity, until their hardware was removed or at least 2 years.

**Results:**

Two hundred thirty-six patients were assessed for eligibility. Of the 282 treated knees which met the criteria for final assessment 55 (19.5%) were unsuccessful. Complications were few but included infection and metal-work prominence. Procedures that were less likely to be successfully included growth disturbances following trauma (18.8% failure) or infection (40%), tumour (66.6%), mucopolysaccharidoses type I (15.7%), spondyloepiphyseal dysplasia (25%) or Blount’s disease (60%). Idiopathic angular deformity showed an 89.5% success rate with guided growth.

**Conclusion:**

In our hands, guided growth had an 80.5% success rate when all diagnoses were considered. We continue to advocate the use of guided growth as a successful treatment option for skeletally immature patients with limb deformity however caution should be employed when considering its use in certain patient groups.

Level of Evidence: Level III, retrospective cohort study.

## Introduction

Angular deformity of the knee is a common phenomenon that can result in pain, gait disturbance, cosmetic deformity and joint degeneration. It has multiple aetiologies with idiopathic being most common as well as physeal disruption secondary to trauma or infection and in various syndromes and skeletal dysplasias. In the past, the standard treatment for correcting angular deformities has been with the use of staples, corrective osteotomy or Bowen’s angular epiphysiodesis [1]. These relatively major interventions can be avoided by using guided growth via a temporary hemiepiphysiodesis with a tension band plate.

Whilst the concept of a hemiepiphysiodesis using a tension band plate (TBP) was used in the early 2000s, it was later reported on by Stevens et al. [3], and his technique became the established method to correct frontal plane deformity around the knee due to its simplicity, safety and low rate of complications [4]. Many studies have shown the TBP’s advantages [3, 5–8] over an osteotomy, faster insertion and removal times when compared to stapling [9], and has also been shown as more suitable than staples in patients less than 8 years due to a partially unossified epiphyses in this cohort [7, 10]. In patients with angular deformity, the mechanical axis will deviate either medially or laterally to a varying degree and the goal of treatment is to normalise these deviations in order to avoid permanent abnormality and morbidity of the adjacent joint surfaces [11].

Our initial experience with guided growth was overall positive, but with more extensive experience we have had a number of patient limbs who failed to respond as anticipated with temporary hemiepiphysiodesis. This prompted us to review our results with the aim of evaluating the efficacy of guided growth surgery involving the distal femur, proximal tibia or both, in order to identify patients who may not benefit from this procedure.

## Materials and methods

We performed a retrospective review of prospectively collected records of all patients undergoing guided growth surgery for coronal plane angular deformity of the knee using the tension band plate at our urban tertiary national referral centre from January 2007 to September 2023. Ethical approval was waived from the local institutional review board.

To be considered for enrolment in this study, patients had to have a coronal plane angular deformity of the knee treated by tension band plating with all having at least one preoperative full-length standing anteroposterior weight-bearing radiograph of the affected limb, as well as post-operative radiographs. Timing between radiographs depended on the treating surgeon. Two different types of two-hole plates were used throughout the study period; the 8-plate by Orthofix (Lewisville, TX, USA) and the Pediplate by Orthopediatrics (Warsaw, IN, USA). The authors gradually moved towards the exclusive use of the Pediplate during the study period. Inclusion criteria were the following: (1) any child with an open physis confirmed by radiographs, (2) diagnosed with coronal plane angular deformity by examination of the lower limb mechanical axis on full-length standing anteroposterior radiographs with any underlying diagnosis, (3) who underwent hemiepiphysiodesis of either the distal femur, proximal tibia or both, (4) patients with follow-up until at least skeletal maturity or removal of metalwork, (5) complete medical data and records available.

Preoperatively, radiographs allowed us to identify the location of the deformity, this allowed us to decide whether treatment should be directed toward the distal femur, the proximal tibia, or both. All patients had a radiographic deformity with the majority displaying clinical deformity. In a small number of patients without clinical deformity but mechanical axis deviation on their radiographs, we used pain as an indication to proceed to surgery.

We assessed the treatment of an individual knee undergoing guided growth surgery. If a patient had a tension band plate placed in one or two physes around the knee (e.g. distal femur or proximal tibia), it still only amounted to one knee treatment. Each time a physis was operated on with a tension band plate and followed up, it was included as an individual treatment. If a patient underwent guided growth surgery, which was followed until skeletal maturity, but during this period sustained a recurrence of deformity, and subsequently underwent a second guided growth surgery, this was identified as two separate guided growth procedures as the first treatment was a success, albeit followed by a recurrence or ‘rebound’ deformity. If a patient had bilateral deformity correction then they were classified as two separate treatments. Clinicians will be familiar with asynchronous correction whereby one leg corrects quickly whilst the other less so or not at all. For this reason, we use the term knee or leg to represent an individual treatment.

Outcomes were decided using a variety of measures which included analysis of weight-bearing radiographs of the lower extremities. If post-operative radiographs were performed they were always weight-bearing in the anteroposterior plane, and only performed if necessary to evaluate persistent clinical deformity. Those performing analysis included consultant orthopaedic surgeons as well as orthopaedic residents. This method of analysis where practitioners independently review and perform digital measurements of radiographs has been shown to be reliable for both inter- and intra-observer variability when assessing paediatric orthopaedic parameters [13], as well as clinical assessments such as inter-malleolar distance or inter-condylar distance. The presence of a limb length discrepancy was also assessed and recorded if present. Mechanical axis deviation (MAD) was assessed and only recorded if both pre and post-operative radiographs were available.

All patient medical records were reviewed throughout the post-operative period as well as at the final follow-up in 2023. The primary diagnosis or underlying aetiology of the angular deformity was recorded as was the patient’s age on the day the plate was inserted as well as the age at which the plate was removed (if applicable). We noted the patient demographics and reviewed all relevant radiographs, as well as patient chart data describing peri-operative experience and outcome. All patients were followed for at least 2 years until skeletal maturity or until their hardware was removed.

A similar surgical technique was used in all cases in line with the methods described by Stevens [3] and evolved by five senior paediatric orthopaedists. The senior author’s current method is as follows: two parallel K-wires are inserted percutaneously parallel to the physis in the epiphysis and metaphysis, and their position is confirmed using fluoroscopy. An incision is made between the guide wires. The periosteum is infiltrated with bupivacaine to reduce post-operative pain. An appropriately sized plate is selected and contoured if necessary. Each screw hole is drilled to a depth of 5 mm. A fully threaded self-tapping cannulated screw (3.0/4.5 mm diameter) of appropriate length is then inserted over each guidewire. The position of the plate and screws is then confirmed on fluoroscopy. Postoperatively the patient is encouraged to weightbear as tolerated.

**Fig. 1:**
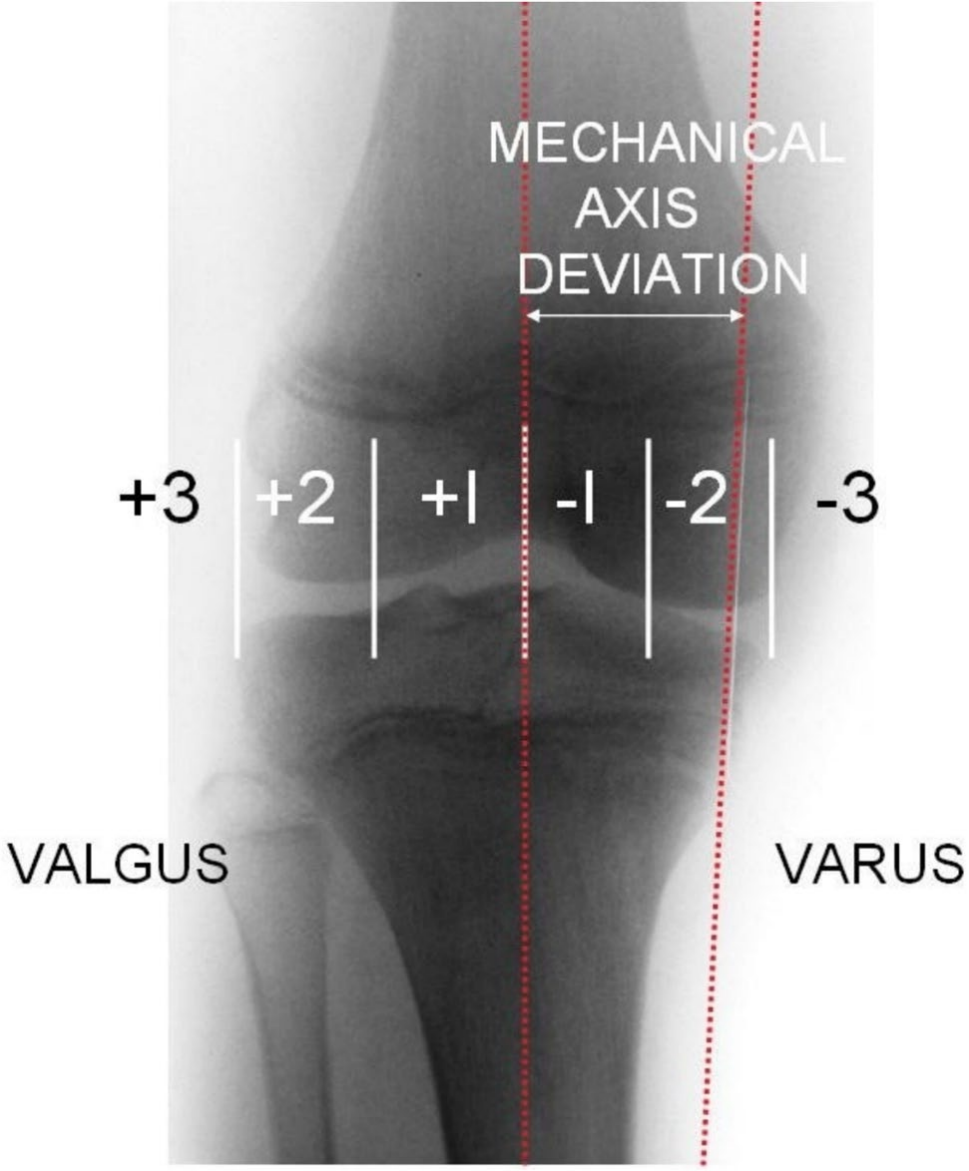
Mechanical axis zones [27]

Alignment analysis was compared preoperatively to those post-operatively and patients were followed up in the outpatient clinic at intervals dictated by their clinical progression. Successful treatment was a radiographic mechanical axis intersecting an area between medial zone 1 to lateral zone 1, which is regarded as physiological and requiring no further treatment^14 15^, with an axis deviation into either zone 2 or 3 either medially or laterally being regarded as an indication for surgical intervention. Once neutralisation of the mechanical axis was achieved, the plates and screws were removed unless the physis was closed and the patient or their next of kin opted not to undergo hardware removal. Any patient who had a persistent clinical deformity or did not have a mechanical axis intersecting medial zone 1 to lateral zone 1 on radiographs in the outpatient department assessed by the senior authors, was offered further surgery in the form of an osteotomy as appropriate (Fig. 1).

On review of all records by the authors at the conclusion of the study period we used the location of the centre of the mechanical axis as defined by Muller and Muller-Farber [16] (zones − 3, − 2, − 1, 0, 1, 2, 3) previously employed by Stevens et al. as the primary outcome measure of treatment success. Any patient that went on to have an osteotomy afterward was regarded as a treatment failure or in the case they declined further surgery a failure was deemed an incomplete correction as evaluated by clinical assessment or + 1 to − 1 zones of mechanical axis. Secondary outcomes were any complications that occurred including pain, screw loosening, hardware failure or breakage, decreased range of motion (defined as < 90° knee flexion within 4 weeks of surgery) or infection. Early infection was defined as within four weeks of surgery.

### Statistical analysis

Statistical analysis was performed using Stata release 17 (Stata Corp. LLC, TX, USA). Descriptive statistics are reported as means with standard deviations (SD) and 95% confidence interval (CI) or range. Simple logistic regression was used to compare outcomes (treatment success vs failure) between diagnosis subgroups and post-estimation analysis was performed to obtain average predicted probability values for treatment success. A *p* value of < 0.05 was considered statistically significant.

## Results

### Demographics

Between 2007 and 2023, 236 (302 physes) patients underwent guided growth surgery using a tension band plate for coronal plane angular deformity of the knee (distal femur and proximal tibia) at our centre. Data on 282 physes (193 patients) was included. To be included, all patients must have been followed for at least 2 years, to skeletal maturity (if this was less than 2 years) or until metalwork was removed; hence, 43 patients were excluded (Table 1). Patients included in our study had a diverse range of aetiologies but all had coronal plane angular deformity of the knee as measured by mechanical axis deviation on AP radiographs. The full range of diagnoses is shown in Table 2. Over 55% percent of patients were male and the mean age at surgery was 10.1 years (SD 3.5, range 1.7–17.3); the mean implant time was 16.2 months (SD 10.3, range 2–63); the mean follow-up time was 5.7 years (range 2.4–9.3).

**Table 1:** Demographics and outcomes

	No. of patients	%
Total no of patients	193	
Total no. of knees (procedures)	282	
Male/female	106/87	55.1%/44.9%
Mean age at surgery	10.1	
Successful	227	80.50%
Failures	55	19.50%
Mean age of failures at surgery	9.3	
Complications	21	7.40%
Recurrences documented	12	4.30%

**Table 2:** Primary diagnosis

	No. of patients	%
Idiopathic	114	40.4
Hurler’s syndrome	38	13.5
Deformity post-trauma	16	5.7
Spondyloepiphyseal dysplasia	12	4.3
Deformity post-infection	10	3.5
Congenital fibular hemimelia	10	3.5
Achondroplasia	9	3.2
Trisomy 21	9	3.2
Osteogenesis Imperfecta	5	1.8
Juvenile idiopathic arthritis	5	1.8
Blount’s disease	5	1.8
Hereditary multiple exostosis	5	1.8
Rickets	5	1.8
Pseudoachondroplasia	4	1.4
Ellis Van Creveld syndrome	3	1.1
Deformity post-tumour	3	1.1
Cerebral palsy	3	1.1
Neurofibromatosis type 1	3	1.1
Spina bifida	3	1.1
Arthrogryphosis	2	0.7
Sickle cell disease	2	0.7
Greig syndrome	2	0.7
Hypochondroplasia	2	0.7
Marfan syndrome	2	0.7
Others*	10	3.5

*Others include proximal femoral focal deficiency, tuberous sclerosis, idiopathic hemihypertrophy, transverse myelitis, Ehlers-Danlos, Kabuki syndrome, multiple epiphyseal dysplasia, neurofibromatosis type 2, Stickler syndrome, Shwachman-Diamond syndrome (all one case)

Idiopathic deformity was the commonest patient cohort accounting for 114 knees (40.4%), (mean age of 11.9 years); Hurler’s syndrome (mucopolysaccharidosis type I) 38 knees (mean age 10.1 years) (13.5%); fracture related deformity in 16 knees (5.7%) (mean age 8.7 years). All other patient groups accounted for 12 or fewer knees.

The number of patients included and their diagnosis are represented in Table 2 and further subgrouped into 3 categories in Table 3.

**Table 3:** Failures by condition [30]

	No. of failures	%
Idiopathic angular deformity	12 in 114	10.50%
Hurler’s syndrome	6 in 38	15.70%
Deformity post-trauma	3 in 16	18.80%
Spondyloepiphyseal dysplasia	3 in 12	25%
Post-infection	4 in 10	40%
Achondroplasia	3 in 9	33.30%
Trisomy 21	3 in 9	33.30%
Juvenile idiopathic arthritis	2 in 5	40%
Blount’s disease	3 in 5	60%
Pseudoachonroplasia	2 in 4	50%
Ellis Van Creveld	2 in 3	66.60%
Deformity post-tumour	2 in 3	66.60%
Hereditary multiple exostosis	2 in 2	100%
Sickle cell w/ deformity	2 in 2	100%
Greig syndrome	2 in 2	100%
Proximal femoral focal deficiency	1 in 1	100%
Tuberous sclerosis	1 in 1	100%
Hemihypertrophy, post-tibial lengthening	1 in 1	100%
Transverse myelitis	1 in 1	100%

### Outcomes

In all, 227 knees (80.5%) were corrected to standard alignment with a mechanical axis between medial zone 1 to lateral zone 1. There was a total of 55 failures (19.5%) across all conditions where patients failed to achieve a mechanical axis to the physiological normal zone. The mean age at surgery of the patients who failed treatment was 9.8 years (SD 3.4, range 3.8–14.4). Table 4 shows the number of failures per condition.

**Table 4:** Subgroup outcomes

	Idiopathic	Congenital	Physis disruption	Total
Failure	13	26	16	55
	11.30%	21.49%	34.78%	19.50%
Correction	102	95	30	227
	88.70%	78.51%	65.22%	80.50%
Total	115	121	46	282
	40.78%	42.91%	16.31%	100%

### Outcome by condition

For idiopathic angular deformity, which made up the most common aetiology in our cohort, there were 12 failures in 114 knees which equates to an 89.5% success rate. Hurler’s syndrome was the second largest patient cohort treated accounting for 6 failed treatments out of 38 (84.3% success). Fracture-related deformity had 3 failures in 16 patients (81.82% success) whilst in 12 patients with a diagnosis of spondyloepiphyseal dysplasia there were 3 failures (75% success). See Table 3 for complete results by condition.

### Outcomes by subgroup

For the purpose of logistic regression, all diagnoses were compiled into deformity attributed to three subgroups—‘idiopathic’, ‘congenital’ and ‘physeal disruption’. The congenital group was made up of patients with achondroplasia, pseudochondroplasia, cerebral palsy, arthrogryphosis, congenital fibular hemimelia, Ehlers-Danlos syndrome, Ellis-Van-Creveld syndrome, Greig syndrome, hemihypertrophy, Hurler’s syndrome, multiple hereditary exostosis, neurofibromatosis types 1 and 2, osteogenesis imperfecta, spina bifida, spondyloepiphyseal dysplasia, Stickler’s syndrome, Shwachman-Diamond syndrome, tuberous sclerosis and trisomy-21. The physeal disruption group was comprised of patients with coronal plane angular deformity following trauma or infection, after tumour, Juvenile Idiopathic arthritis, sickle-cell disease and also those with Rickets and Blount’s disease. Finally, the idiopathic group contained patients whose deformity could not be attributed to any underlying condition (one further patient with a diagnosis of transverse myelitis was added to the idiopathic group). Table 4 summarises treatment outcomes between the 3 deformity subgroups.

Logistic regression was performed to analyse the relationship between the three diagnosis subgroups and treatment success. It was found that the odds of treatment success decreased by 53% in congenital patients and by 76% in patients with physeal disruption when compared to patients in the idiopathic group. These results are illustrated in detail in Table 5.

**Table 5:** Results of simple logistic regression analysing the relationship between treatment success and diagnosis subgroup

Diagnosis	Odds ratio	Standard error	*z*	*P* > *z*	95% confidence interval (CI)	
Idiopathic	1	(BASE)				
Congenital	0.47	0.17	− 2.07	0.04	0.23	0.96
Physis disruption	0.24	0.1	− 3.35	0	0.1	0.55
*Constant*	7.85	2.31	7	0	4.41	13.97

**Fig. 2:**
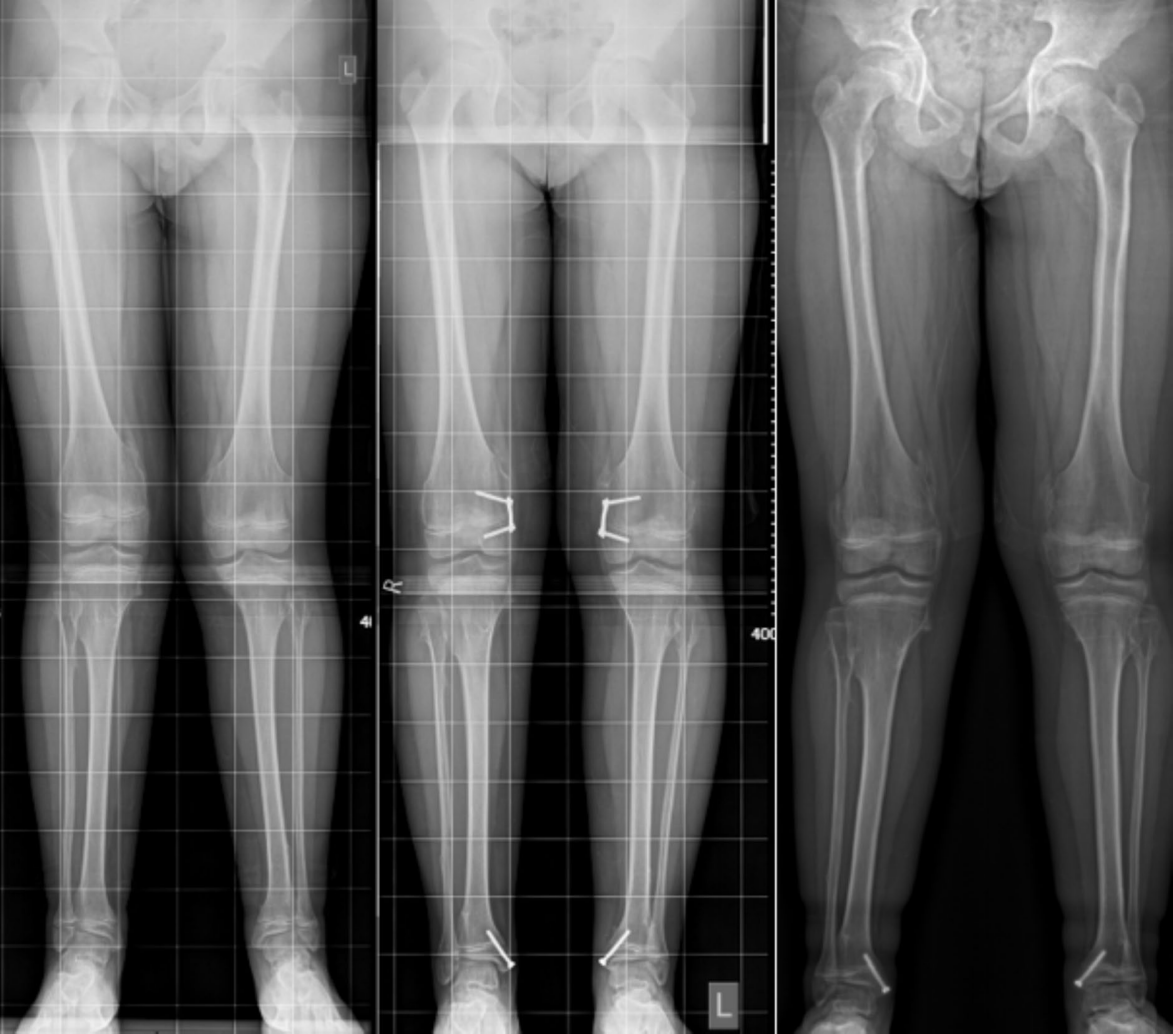
Radiographs of a patient pre-, during and post-coronal plane angular deformity correction

Post-estimation analysis using marginal means revealed average predicted probability (of treatment success) scores of 89% (CI 83–94), 79% (CI 71–86) and 65% (CI 51–79) in idiopathic, congenital and physis disruption groups respectively. These results are summarised in Table 6 and a visual representation is illustrated in Fig. 2.

**Table 6:** Post-estimation analysis results of simple logistic regression analysing the relationship between treatment success and diagnosis subgroup

Diagnosis	Margin (average predicted probability)	Standard error	*z*	*P* > *z*	95% confidence interval (CI)	
Idiopathic	0.89	0.03	30.04	0	0.83	0.94
Congenital	0.79	0.04	21.03	0	0.71	0.86
Physis disruption	0.65	0.07	9.29	0	0.51	0.79

### Complications

There were 32 complications documented at the final follow-up: hardware-related pain = 6; recurrences = 11; post-operative infection = 2; hardware failure = 8; overcorrection = 2; contracture deformity = 1. See below for detailed information regarding complications (Table 7).

**Table 7:** Mean age range per group

Diagnosis	Mean age	Range
Idiopathic	11.9	7.0–16.0
Achondroplasia	8.4	7.0–11.0
Hurler’s syndrome	10.1	6.0–14.0
Infection	7	7.0–14.0
Blount’s disease	9.7	4.0–14.0
Congenital fibular hemimelia	13.6	5.0–14.0
Post-trauma	8.7	4.0–15.0
Rickets	11.5	10.0–15.0
Spondyloepipheseal dysplasia	8.6	4.0–13.0

### Pain

**Fig. 3:**
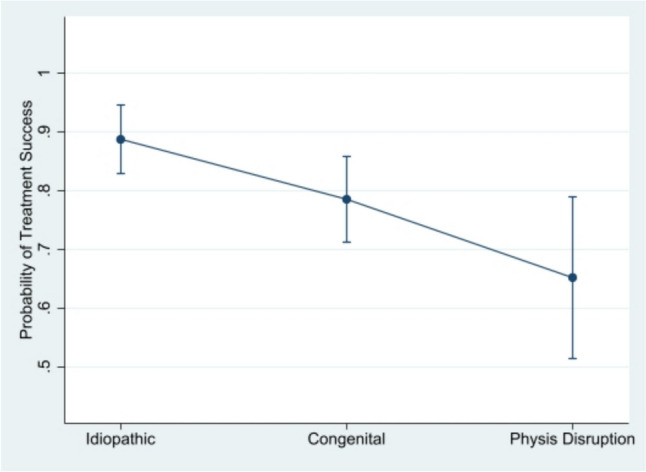
Average predicted probability of treatment success by diagnosis subgroup with 95% confidence intervals

Six patients had hardware-related pain following treatment: one patient with congenital fibular hemimelia, one with Hurler’s syndrome, one patient with trisomy 21, one patient with spondyloepiphyseal dysplasia and two patients with idiopathic angular deformity. One of the idiopathic knees required two steroid injections for their symptoms which resolved. One patient with spondyloepiphyseal dysplasia (failed treatment) required a release of tethered tissue which presented as pain at the plate site, whilst one patient with trisomy 21 reported pain whilst plates were in situ Fig. 3.

### Recurrence

Two-hundred eighty-two knees had an initial successful coronal plane deformity correction after their index procedure; however, 11 knees went on to develop a recurrence of the deformity. The highest number of recurrences occurred in knees with a fracture-related deformity, of which there were 3. There were two recurrences in knees with idiopathic deformity, congenital fibular hemimelia and Hurler’s syndrome respectively, whilst there was one recurrence in hereditary multiple exostosis and one in a patient with infection-related deformity. Of note, the recurrences in congenital fibular hemimelia were in the same patient who underwent 3 successful guided growth procedures of the same knee which recurred twice (Table 8).

**Table 8:** Recurrence by subgroup

Original deformity diagnosis	No. of recurrences
Fracture-related deformity	3
Idiopathic deformity	2
Congenital fibular hemimelia	2
Hurler’s syndrome	2
Infection related deformity	1
Hereditary multiple exostosis	1

### Infection

There were 2 infections in the post-operative period (1 patient with a diagnosis of hereditary multiple exostosis and 1 with neurofibromatosis type 1 with anterolateral bowing both of which had successful guided growth surgery). Both had a superficial wound infection following routine removal of hardware and were treated successfully with a short course of antibiotics.

### Hardware failure

There were 8 documented hardware failures. Screw loosening occurred in 3 patients (2 with Hurler’s syndrome and 1 with neurofibromatosis type 1). Another Hurlers’ patient complained of hardware prominence. Among patients with idiopathic deformity, one patient suffered plate and screws backing out causing pain, whilst one needed a revision procedure for a loose screw. Another patient with neurofibromatosis type 1 suffered screw loosening whilst a further patient with spondyloepiphyseal dysplasia required a revision for screw loosening and another procedure to release tethered tissue.

### Miscellaneous

One patient suffered a contracture deformity following failed treatment (Hurler’s syndrome), whilst two patients with idiopathic deformity were overcorrected. One patient with spondyloepiphyseal dysplasia developed knee instability following successful treatment requiring surgery.

## Discussion

### Summary of findings

This study includes data on 282 knees undergoing guided growth hemiepiphysiodesis surgery using tension band plates for coronal plane angular deformity. Overall, 80.5% of deformities were corrected to standard alignment mechanical axis in the physiological normal range [15] with 92.9% of patients having at least 2-year follow-up. Our definition of success was similar to those of Stevens [3] and Burghardt [5] where success was defined as complete deformity correction to acceptable mechanical alignment or avoiding the need for corrective osteotomy. We did not analyse the mechanical lateral distal femoral angle and mechanical medial proximal tibial angle as this was not routinely measured during the follow-up period. If guided growth surgery did not correct the deformity clinically or radiographically then the treatment was regarded as a failure. In the case of failed treatment, an osteotomy was recommended however not all patients and their families opted for further surgery.

### Literature review

To our knowledge, this is the largest single-centre cohort study in the literature reporting on guided growth using the tension band plate. We have included more results on pathologic physes than the largest studies on the topic reported previously [17]. Our patient groups were so diverse in terms of aetiology therefore grouping them into specific categories seems inappropriate.

Published reports have shown varied outcomes. Burghardt [5] et al. had a 90% success rate in their series, Danino et al. reported a 70% correction rate with guided growth [18] whilst Funk et al. reported a 57.9% failure rate in their 38 knees [19]. Our results are comparable to the more successful reports with this procedure perhaps due to the high volume of patients we see with deformity around the knee. In this study, patients with idiopathic deformity have the most predictable outcomes with tension band plates and showed an 89.5% success rate, falling below previously reported success rates as high as 99.6% [20] and 100% respectively [12, 20, 21].

There were poorer outcomes (> 15% failure) in patients with Hurlers syndrome, infection or fracture-related deformity, spondyloepiphyseal dysplasia, trisomy 21, Blount’s disease and dwarfism (pseudochondroplasia and achondroplasia) in line with previous studies where pathological cases had a lower success rate than those with idiopathic deformity [12, 20]. In cases of trauma or infection where there was a possibility of a physeal bar then appropriate higher-order imaging such as MRI and CT were carried out. It would now be the recommendation of the authors that the presence of a bar from any cause is a contraindication to the use of guided growth. When subgrouped under three main categories, patients with deformity related to physeal disruption showed a 34.8% failure rate which is disappointing. Whilst it could be argued that guided growth surgery is still worth attempting in these pathologies due to the low associated morbidity, it further reinforces the importance of counseling patients and their families around this in order to manage their expectations. Many patients may deem a smaller procedure with a 65% chance of successful outcome more acceptable than a larger procedure.

It has been shown that patients with Blount’s disease are more likely to have surgical failure [12]. In a systematic review of 63 hemiepiphysiodesis procedures in patients with Blount’s disease, Fan et al. reported a 51% success rate [22], whilst Oto et al. had a 64% successful outcome [23]. This compares with our 40% success rate however it is difficult to extrapolate any meaningful finding from our cohort as it only accounted for 5 out of 282 physes treated. Our results are in keeping with those of Schoerlucke et al. [24]. It has been reported that deformity in infantile Blount’s disease should be corrected before age 4 [12]. The mean age at surgery in our Blount’s cohort was 9.5 years, perhaps explaining the 60% failure rate in the five patients we treated. However, we cannot form any useful conclusion regarding the management of Blount’s disease given the relative rarity of this condition in our practice. In patients with pseudochondroplasia, we had a success rate of 50%, similar to other studies [12] and slightly lower than those reported by Oto et al. [23]. Our Dwarfism cohort had a 38.5% failure rate, perhaps due to the low growth potential in this group rendering them unsuitable for guided growth.

Whilst age at surgery was not our primary outcome when performing our study, it has been shown that correction is faster in children aged less than 10 years compared with those older than 10 years [8] and patients with at least 3 years of growth at the time of plate implantation have a better chance of achieving the surgical aim [26. The mean age of our group was 10.1 years at surgery, but interestingly the mean age at surgery of our patients who failed treatment was 9.8 years. High body mass index has been shown to have a negative impact on success rates [12] with TBPs but we did not record it in all of our patients so have not reported on it. Patient sex has been shown not to influence the success of treatment so we did not focus on it here [25] nor was it our aim to assess correction rates for valgus or varus knees individually as this has also been shown to be similar [25].

### Complications

We report 32 (11.3%) complications over 18 years using this treatment method, with our 2.8% rate of hardware failure being slightly less than those reported in previous studies [26]. The low complication rate further justifies employing the use of tension band plates even in patient groups where their diagnosis has shown to be a negative prognostic marker due to its lower associated morbidity than an osteotomy [12] as long as the patients’ family are aware of its decreased efficacy in these cases.

### Limitations

There are several limitations to our study we would like to acknowledge. This is a retrospective review albeit of prospectively collected data hence we were relying on the quality of notes recorded in the chart for the relevant clinical information on decision making. During chart review we did seek to assess body mass index data however whilst the patients’ weight is always recorded, their height is not hence we were unable to estimate its impact on treatment failure therefore excluded it as a parameter.

## Conclusion

Whilst predictable successful outcomes can be expected in idiopathic deformity, we recommend caution when using tension band plates to treat angular deformity of the knee in patients with deformity following infection or trauma, spondyloepiphyseal dysplasia, Blount’s disease, dwarfism and Hurler’s syndrome. There is a low associated morbidity with tension band plates in those performing high volume of this procedure.

## Declarations

The Ethics Committee of the Children’s Health Ireland waived the need for ethics approval and the need to obtain consent for the collection, analysis, and publication of the retrospectively obtained and anonymised data for this study. All results were given a unique anonymous identifier.
